# Changes in Breath Trihalomethane Levels Resulting from Household Water-Use Activities

**DOI:** 10.1289/ehp.8171

**Published:** 2005-11-15

**Authors:** Sydney M. Gordon, Marielle C. Brinkman, David L. Ashley, Benjamin C. Blount, Christopher Lyu, John Masters, Philip C. Singer

**Affiliations:** 1 Battelle Memorial Institute, Columbus, Ohio, USA; 2 National Center for Environmental Health, Emergency Response and Air Toxicants Branch, Centers for Disease Control and Prevention, Atlanta, Georgia, USA; 3 Centers for Public Health Research and Evaluation, Battelle Memorial Institute, Durham, North Carolina, USA; 4 Department of Environmental Sciences and Engineering, University of North Carolina–Chapel Hill, Chapel Hill, North Carolina, USA

**Keywords:** biomarkers, breath analysis, disinfection by-products, exposure, trihalomethane, water use

## Abstract

Common household water-use activities such as showering, bathing, drinking, and washing clothes or dishes are potentially important contributors to individual exposure to trihalomethanes (THMs), the major class of disinfection by-products of water treated with chlorine. Previous studies have focused on showering or bathing activities. In this study, we selected 12 common water-use activities and determined which may lead to the greatest THM exposures and result in the greatest increase in the internal dose. Seven subjects performed the various water-use activities in two residences served by water utilities with relatively high and moderate total THM levels. To maintain a consistent exposure environment, the activities, exposure times, air exchange rates, water flows, water temperatures, and extraneous THM emissions to the indoor air were carefully controlled. Water, indoor air, blood, and exhaled-breath samples were collected during each exposure session for each activity, in accordance with a strict, well-defined protocol. Although showering (for 10 min) and bathing (for 14 min), as well as machine washing of clothes and opening mechanical dishwashers at the end of the cycle, resulted in substantial increases in indoor air chloroform concentrations, only showering and bathing caused significant increases in the breath chloroform levels. In the case of bromodichloromethane (BDCM), only bathing yielded a significantly higher air level in relation to the preexposure concentration. For chloroform from showering, strong correlations were observed for indoor air and exhaled breath, blood and exhaled breath, indoor air and blood, and tap water and blood. Only water and breath, and blood and breath were significantly associated for chloroform from bathing. For BDCM, significant correlations were obtained for blood and air, and blood and water from showering. Neither dibromochloromethane nor bromoform gave measurable breath concentrations for any of the activities investigated because of their much lower tap-water concentrations. Future studies will address the effects that changes in these common water-use activities may have on exposure.

The trihalomethanes (THMs) chloroform (CHCl_3_), bromodichloromethane (BDCM), dibromochloromethane (DBCM), and bromoform are major by-products of water-disinfection processes involving chlorine [International Agency for Research on Cancer (IARC) 1991]. Typical household activities involving chlorinated water, such as showering, bathing, washing dishes, or drinking tap water, expose individuals to the THMs by inhalation, dermal contact, or ingestion. Such exposure varies from person to person and depends on the individual’s water use. A number of studies have found an association between elevated levels of THMs in drinking water and adverse health effects, including bladder (Cantor et al. 1987, 1998; Morris et al. 1992; Vena et al. 1993) and rectal (Hildesheim et al. 1998; Morris et al. 1992) cancers, and birth defects (Bove et al. 1995; Graves et al. 2001).

Common household activities such as showering, bathing, drinking water, and washing clothes and dishes are potentially important contributors to THM exposure (Nieuwenhuijsen et al. 2000; Wallace 1997). Inhalation and dermal contact resulting from showering and bathing have been shown to be significant routes of exposure to THMs (Ashley and Prah 1997; Backer et al. 2000; Miles et al. 2002; Wallace 1997; Weisel and Jo 1996; Weisel et al. 1999). Backer et al. (2000) exposed subjects to THMs in tap water under controlled conditions through ingestion, showering, and bathing, and measured blood concentrations before and after exposure. The levels of the three measurable THMs increased sharply as a result of showering or bathing, but drinking 1 L of tap water resulted in only a small increase. Several other studies have also measured the uptake of THMs from various water-use activities in body fluids, including blood, exhaled breath, and urine. However, the contribution of such activities to the speciation and concentration of THMs in body fluids has not been studied comprehensively. This information would allow us to reliably apportion the contributions of these activities to overall THM exposure and improve the interpretation of data collected in such studies.

Most previous determinations of the uptake of CHCl_3_ and other THMs by dermal absorption, inhalation, or ingestion resulting from showering, bathing, or drinking water have been based on measurements of exhaled breath (Benoit et al. 1998; Gordon et al. 1998; Jo et al. 1990a, 1990b; Levesque et al. 2002; Wallace 1987, 1997; Weisel and Jo 1996; Weisel et al. 1992, 1999); somewhat fewer have used venous blood (Ashley and Prah 1997; Backer et al. 2000; Lynberg et al. 2001; Miles et al. 2002). The Total Exposure Assessment Methodology (TEAM) study that was conducted between 1979 and 1984 provided a major body of data on THM concentrations at consumers’ taps and was also the source of most measurements of personal exposures to airborne CHCl_3_ (Wallace 1987). The TEAM study indicated, for example, that indoor residential air contributed 25–30% of the combined air–tap-water daily intake of CHCl_3_ and BDCM (Wallace 1997). Chloroform levels measured in breath after showering have also been found to be correlated with their concentrations in the shower water and air (Jo et al. 1990a; Weisel et al. 1999) and with the time spent carrying out the activity (Gordon et al. 1998).

Although blood levels are generally more sensitive to low exposures (Churchill et al. 2001; Miles et al. 2002), there are distinct advantages in carrying out measurements of volatile organic compounds (VOCs) in breath. Chief among these are that breath analysis is noninvasive and is therefore usually more acceptable to human subjects, resulting in higher levels of participation in exposure studies. Additionally, because the breath sample supply is virtually limitless, it allows the rapid collection of multiple samples and even lends itself to continuous real-time monitoring (Gordon et al. 1998). Because of the dynamic equilibrium between the concentration of a VOC in the blood and its concentration in exhaled breath (Wallace et al. 1996), breath measurements can be used to estimate body burden and to detect changes in body burden with time (Gordon et al. 1992; Wallace et al. 1993; Weisel et al. 1992).

The present study was undertaken to examine, through exhaled-breath measurements, which common household water-use activities lead to an increase in the internal dose levels of CHCl_3_ and the other potentially harmful THMs in the human body. This will inform future studies by indicating which activities should be investigated more thoroughly. Water, air, blood, and breath samples were collected from subjects for each activity that was expected to elevate the internal dose of the THMs. An overview of all of the methods used in this study and a summary of the results obtained have been published by Nuckols et al. (2005). In this article, we show more clearly which activities are the major determinants of exhaled-breath THM concentrations, and focus on some of the correlations that are associated with these activities.

## Materials and Methods

The experimental approach and methods used in this investigation have been presented elsewhere (Nuckols et al. 2005), so only a brief summary is given here. Emphasis is placed on those aspects that are particularly germane to this study.

### Study locations and participants.

The study was conducted in two single private homes, one in North Carolina (NC) and the other in Texas (TX). The selection of these locations was based upon the assumption that the distribution of THMs in the public water supplies that served these areas would be different because of anticipated high bromide concentrations in the TX raw water source. However, instead of the dominant bromine-containing species that were expected at the TX site, relatively low concentrations of BDCM, DBCM, and bromoform were found, probably due to heavy rains in the area during the week preceding the subject activities in the home, which diluted the bromide concentration in the raw water. Chloroform was the dominant THM species, and overall THM levels were less than expected.

Both homes were single-story, ranch-style residences (about 1,200 ft^2^ or 111.5 m^2^ total floor area) with three bedrooms, two bathrooms, and central heating, ventilating, and air conditioning (HVAC) systems. Tap water was provided in each case by the local public utility, which used combined chlorine as the residual disinfectant. The measured free chlorine residuals in the test homes at the time of sample collection were consistent with standard practice for chloraminated systems. THM levels at other locations in the distribution systems for the two utilities during the periods of this study were assumed to be similar because continuing THM formation in distribution systems is quenched in the presence of combined chlorine. The thermostat in each house was set at 75°F and the HVAC fan was set to the “on” position during the entire study period. The exhaust fan in the study bathroom was turned off throughout the study.

Seven healthy, young subjects, 21–30 years of age, were enrolled into the study: four in NC (three males and one female) and three in TX (one male and two females). Two of the males at the NC site reported their race as African American; all other participants reported their race as Caucasian. All subjects were nonsmokers and had a body mass index of 22–24. The study protocol was reviewed and approved by the institutional review boards of the Centers for Disease Control and Prevention (CDC) and the Battelle Memorial Institute; informed written consent was obtained from the subjects before they took part in the study.

### Exposure regimen.

To avoid inadvertent exposure to disinfection by-products, the subjects slept in the study residence the night before exposure activities began. Each subject took part in several common water-use activities over 2 distinct days that were roughly 1 week apart. The prescribed water-use activities are summarized in Table 1. Activities each day were separated by at least 1 hr (3 hr after showering and bathing) to ensure that blood and breath THM concentrations returned as close as possible to baseline levels before the start of the next exposure activity. Each activity was preceded and followed at fixed times by the collection of tap water and blood samples. To ensure privacy, each subject wore a bathing suit for the showering and bathing activities. Baseline indoor air and breath samples were collected at the start of each day before the subjects undertook any prescribed activities. Air samples then were collected during each activity, whereas breath samples were collected 5 min after each activity. Two of the subjects followed a more intensive protocol than the other subjects. These subjects performed the same activities as the other subjects, but additional breath, air, and water samples were collected during their shower activity. The additional samples were taken in an attempt to better define peak air and breath concentrations for the THMs during showering. The THM results obtained with the intensive shower protocol will be reported in a forthcoming article.

### Sample collection and analysis.

#### Shower and bath air.

We collected air samples from the shower stall during each shower event to evaluate inhalation exposure for the subjects. We collected 13 samples for each of the five subjects who followed the standard protocol, and 19 samples for each of the two subjects who followed the intensive shower protocol. Depending on the specific water-use activity (Table 1), we manually collected either time-integrated or short-term (“grab”) air samples into precleaned and evacuated SUMMA 6-L stainless steel canisters (Scientific Instrumentation Specialists, Moscow, ID, and Biospherics, Hillsboro, OR). Grab samples were taken by rapidly opening the canister valve and allowing air to flow into the canister until near-atmospheric pressure equilibrium was attained (< 1 min). To collect time-integrated air samples, we attached precut stainless steel tubes of varying internal diameter and length to each canister. These tubes served as critical flow orifices that regulated the flow rate into the canister and ensured that it filled in approximately the same time as the time taken to complete the specific water-use activity being monitored. Integrated 13-min air samples associated with the showering activity were collected from the start of the showering activity until 3 min after the water was turned off. For the bathing activity, integrated 23-min air samples were collected from the start of the 6-min bath filling period, through the 14-min bathing (subject immersion) period, and ended 3 min after the subject got out of the bathtub.

**Figure 1:**
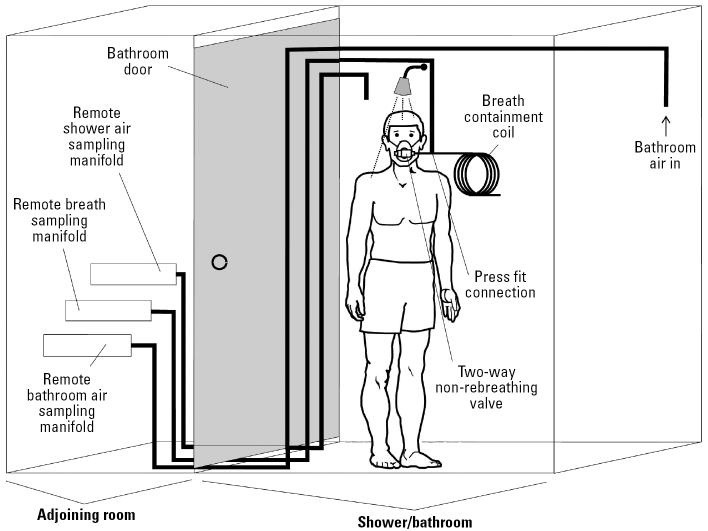
Schematic of breath/air sampling system for intensive shower protocol. For the collection of shower stall air samples, the breath containment coil was removed from the system at the press-fit connection. Breath samples were collected 5 min after the showering activity ended. For consistency with the data from the time-integrated shower stall air samples collected during the standard shower protocol, the air concentrations from the multiple grab samples taken during the intensive shower protocol were averaged.

For the standard shower protocol, the single canister used to collect shower stall air was suspended from the shower curtain rod close to the shower head; for the bath activity, the canister was placed on the bathtub ledge close to the subject’s head. Because of space limitations in the bathrooms and the need to rapidly collect multiple canister grab samples (~ 1–2 min apart) for the intensive shower protocol, three specially constructed stainless steel manifolds were used, one to sample the bathroom air, one to collect the shower stall air, and the third to collect the breath samples (Figure 1). One end of the sampling tube for bathroom air was positioned in the bathroom, and sampling was performed remotely in an adjoining room via a length of stainless steel tubing (0.22 cm i.d., ~ 6.0 m long) attached to the manifold. For the collection of air and breath samples in the shower stall, two separate lengths of stainless steel tubing (0.22 cm i.d., ~ 16 m long) were used. The tube used to collect breath samples was connected to a T-piece that joined the face mask and breath containment coil; the other end was connected to the remote breath sampling manifold located outside the bathroom. Sampling with all three systems involved manual opening of each canister valve in turn and then closing the valve at the end of the sampling period.

The breath containment coil (899 cm × 1.3 cm i.d.), which is based on the alveolar breath sampling device developed by Raymer et al. (1990), provides a sufficiently large buffer volume (1.1 L) that ensures that the breath being sampled is primarily alveolar because it is from the end of the expiration. The canisters were stored at room temperature and, at the end of each sampling day, shipped by overnight express courier to the Atmospheric Science and Applied Technology Laboratory at Battelle (Columbus, OH) for THM analysis.

Samples were analyzed for THMs by automated gas chromatography/mass spectrometry (GC/MS) using a modified version of U.S. Environmental Protection Agency (EPA) Method TO-14 (Winberry et al. 1990). The GC was connected to a cryogenic preconcentration trap, which was cooled to −185°C for sample collection and heated to 120°C during sample desorption. A six-port valve controlled sample collection and injection. The preconcentrator was also equipped with an auto-sampler so that up to 16 canister samples could be analyzed automatically. The sample volume was 90 mL. Analytes were chromatographically resolved on a Restek RTX-1 fused silica capillary column (Restek Corp., Bellefonte, PA). The mass spectrometer was operated in the full scan mode. Target analytes were identified by matching the mass spectra acquired from the sample to the mass spectral library from the National Institute of Standards and Technology (Gaithersburg, MD). Quantification of all identified peaks was based upon multipoint calibration curves, which were generated for each target analyte at the start of the study. During each analysis period, a single-point calibration was run; precision for each THM was typically < 20% relative SD.

#### Exhaled breath.

As shown in Table 1, we monitored eight water-use activities during the subject’s day-1 sampling visit and seven water-use activities during the day-2 visit, approximately 1 week later. We collected a total of 15 breath samples from each of the five subjects who participated in the standard shower protocol, and 22 samples from each of the two subjects who followed the intensive shower protocol. In all cases, except while the subject was in the room adjacent to the operating shower (i.e., secondary shower exposure activity), breath samples were collected 5 min after completion of the activity. The single breath canister (SBC) procedure, developed by Pleil and Lindstrom (1995a), was used to collect the samples, except in the case of the intensive shower protocol. The SBC sampling method is a self-administered procedure in which the subject exhales alveolar air directly into an evacuated 1-L Silcosteel stainless steel canister (Entech, Simi Valley, CA) fitted with a short Teflon tube that serves as a disposable mouthpiece. To obtain a breath sample, the subject closes his or her lips over the open end of the Teflon tube while exhaling, opens the canister valve, and fills the evacuated volume. The subject is instructed to begin sample collection near the end of a normal resting tidal breath to provide what is mostly alveolar breath.

For the intensive shower protocol, the subject was fitted with a face mask (model 8932; Hans Rudolph, Inc., Kansas City, MO) that covered his or her mouth and nose and was equipped with a two-way non-rebreathing valve set. The subject inhaled through the one-way valve in the inhalation port, which was left open to the shower stall air. The exhalation port of the mask was attached via a stainless steel tube to a convoluted polytetrafluoroethylene breath containment coil in an adjoining room (Figure 1). The subject exhaled normally through the one-way valve in the exhalation port. Exhaled breath (primarily alveolar) was collected remotely in an evacuated 1-L Silcosteel canister, attached to the breath sampling manifold by manually opening the canister valve and allowing the breath to flow back through the containment coil, back through the stainless steel sampling line, and into the canister until the contents of the canister reached near-atmospheric pressure (≤1 min). As in the case of the intensive bathroom air samples, the sampling manifold allowed the rapid collection of successive grab breath samples.

Background samples were obtained once each morning before any water-use activities began. Samples were shipped at the end of the day by overnight express courier to Battelle for THM analysis, which was carried out by automated GC/MS as described above. To correct for dilution of alveolar air from dead volume air in the breath sample and to normalize the concentration of each analyte to correctly reflect the breath concentration, we measured the carbon dioxide concentration in each canister sample using a CO_2_ monitor (Pryon model SC-300; Pryon Corp., Menomonee Falls, WI) equipped with an external infrared CO_2_ sensor. Analysis of CO_2_ in the breath provides an accurate correction factor for the approximate amount of alveolar breath (as opposed to whole breath) collected in the sample (Pleil and Lindstrom 1995b).

#### Blood.

Each participant provided a total of 26 blood samples over the course of the 2-day study: 14 on day 1 and 12 on day 2. Samples were taken approximately 5 min before and after each activity (and 30 min after the end of the shower and bath activities), using a venous catheter that remained in the subject’s arm throughout each study day (~ 12 hr). Blood collection (Vacutainer) tubes were specially treated before use to remove background contamination (Cardinali et al. 1995). After collection, samples were refrigerated and then packed into coolers with ice packs for shipping by overnight express courier to the Division of Laboratory Sciences of the CDC (Atlanta, GA) for analysis. Details of these procedures have been described elsewhere (Bonin et al. 2005).

The whole blood samples were analyzed for THMs using solid-phase microextraction GC/isotope dilution MS, with the mass spectrometer operating in the selected ion monitoring mode. Stable isotopically labeled analogs of the compounds of interest were added to the samples as internal standards, and quantification was accomplished by measuring specific ion responses relative to those of the corresponding labeled analogs (Ashley et al. 1992; Cardinali et al. 2000).

#### Water.

We collected a total of 21 (normal shower protocol) or 22 (intensive shower protocol) water samples over each 2-day study period. All water samples were collected in headspace-free 40-mL acid-washed glass vials with screw caps. Residual chlorine was quenched with ammonium sulfate. Besides the samples of water associated with each activity, we collected several samples from a cold water tap during each exposure day to establish baseline THM concentrations. The temperature of the water used in each activity was controlled over a limited range and recorded as samples were collected for each study activity. For the shower activity, the showerhead in the study bathroom of each residence was replaced with a standardized showerhead designed to maintain constant flow; temperature, flow rate, and duration were maintained constant during showering. Water samples, which were collected midway through each activity, were shipped by overnight express courier to the Drinking Water Research Center laboratories at the University of North Carolina–Chapel Hill, School of Public Health, for analysis. THMs were analyzed using a standard liquid–liquid extraction GC/electron capture detection procedure (U.S. EPA 1995). Details of the sample collection and analysis procedures are presented elsewhere (Nuckols et al. 2005; Singer et al. 2003).

### Data analysis.

Before evaluating the air and breath data to determine which common residential water-use activities have the greatest effect on exhaled-breath THM levels, we used Dixon’s outlier test to help identify possible outliers, especially inexplicable extreme values. This analysis resulted in the removal of a single data point from each of the air and breath CHCl_3_ and BDCM data sets for several activities, including those involving hand washing, dishwashing by hand, bathing, and mechanical washing of clothes. Because of problems that occurred during the collection of the air and breath data while conducting the first of the two intensive shower protocols (in NC), the shower activity air and breath data for this subject were rejected. In addition, some of the breath data obtained from one of the standard protocol subjects were discarded because of breath CO_2_ levels much lower than expected, indicating a sample collection problem for this subject. Measured breath and air concentrations that were below the limit of detection (LOD) were assigned a value equal to half the LOD for the calculation of sample means and SDs. Because all of the measured breath concentrations for DBCM (LOD = 0.8 μg/m^3^) and bromoform (LOD = 1.0 μg/m^3^) were below the LOD, data analyses were confined to CHCl_3_ and BDCM.

To adjust for the wide variability in THM water concentrations between the NC and TX sites, we normalized the room air and exhaled-breath data with respect to the water concentrations. The normalized concentrations for the air and breath samples collected before (i.e., baseline) and after each exposure activity for all subjects were compared using the Mann-Whitney *U*-test statistic at a significance level of *p* ≤0.05.

Using the raw measured concentrations, Spearman correlation coefficients were evaluated for pairs of measurements from the breath, blood, air, and water results in post-exposure shower and bath activity samples for all subjects.

## Results

### Effects of tap-water quality and water-use activity on indoor air and exhaled-breath concentrations.

Table 2 summarizes the over-all mean water concentrations for CHCl_3_ and BDCM over all subjects by exposure day and sampling site (NC and TX). A detailed analysis of the data indicates that there were large differences in the THM concentrations over the course of the exposure measurements (Nuckols et al. 2005). Mean water concentrations were much higher on both exposure days at the NC site than in TX for CHCl_3_ as well as for BDCM.

**Figure 2:**
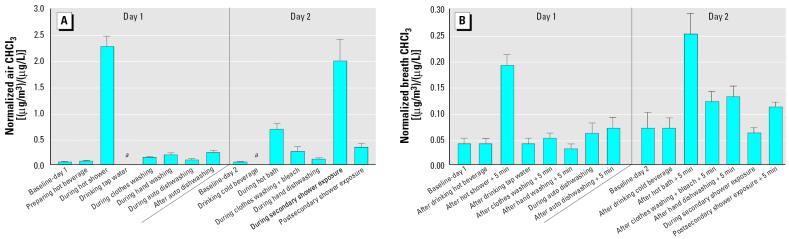
Normalized mean CHCl_3_ concentrations in air (*A*) and exhaled breath (*B*) for subjects at both sampling sites as a function of various common household water-use activities (mean ± SE). All activities in air (except for dishwasher open and postsecondary shower exposure) were measured during the exposure. For all activities, exhaled breath (except during automatic dishwashing and during secondary shower exposure) was measured 5 min after the exposure ended. **a**No air sample was collected for this activity.

The effect of water-use activities on the mean indoor air and exhaled-breath concentrations of CHCl_3_ normalized with respect to tap-water concentrations (micrograms per cubic meter of breath per microgram per liter of water) for all subjects on both exposure days and at both sites is shown in Figure 2. Most indoor air samples for the water-use activities shown on the *x*-axis in Figure 2A were taken during the period of each exposure activity, whereas most exhaled-breath samples shown in Figure 2B were collected 5 min after each exposure activity ended. Compared with the relevant day-1 or day-2 preexposure (baseline) level, the increase in indoor air concentration of CHCl_3_ was greatest for subjects as a group at both sites for the showering and the secondary shower exposure activities (> 40-fold increase), followed by the bathing activity (> 10-fold increase), and post-secondary shower exposure and exposure due to the machine washing of clothes using bleach (> 5-fold increase).

Figure 2B shows the mean normalized exhaled-breath concentrations of CHCl_3_ for the preexposure samples and those collected 5 min after each activity ended. The exhaled-breath levels are approximately an order of magnitude lower than the levels in the indoor air for the corresponding activities. The activities that resulted in marked increases in breath levels over the corresponding baseline level across all subjects at both sites were showering (5-fold increase) and bathing (6-fold increase). The strong increases in relative air concentrations noted above for the secondary shower exposure, the postsecondary shower exposure, and exposure due to the mechanical washing of clothes using bleach were not reflected in the corresponding breath measurements.

**Figure 3:**
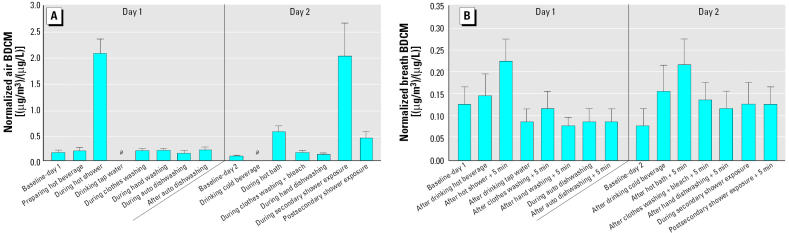
Normalized mean BDCM concentrations in air (*A*) and exhaled breath (*B*) for subjects at both sampling sites as a function of various common household water-use activities (mean ± SE). All activities in air (except for dishwasher open and postsecondary shower exposure) were measured during the exposure. For all activities, exhaled breath (except during automatic dishwashing and during secondary shower exposure) was measured 5 min after the exposure ended. **a**No air sample was collected for this activity.

The mean normalized indoor air and exhaled-breath concentrations of BDCM for the preexposure samples and defined activity samples are shown in Figure 3. Although the concentrations of BDCM in the tap water, and hence in the air and breath samples, were much lower than those for CHCl_3_, these plots are generally very similar to those obtained for CHCl_3_, but with greater variability in the data. Although the air BDCM levels for the showering and secondary shower exposure activities are much greater than their baseline levels (> 10-fold increase in each case), followed by the bathing activity (4-fold increase), only the showering and bathing activities are moderately higher in the breath BDCM measurements (~ 2-fold increase in each case).

### Correlations among breath, blood, water, and air measurements.

Table 3 presents Spearman correlation coefficients for CHCl_3_ and BDCM for blood and breath in samples obtained from the showering and bathing activities undertaken by all subjects. For CHCl_3_ from the shower event, Spearman coefficients are highly significant for breath and air, blood and breath, air and blood, and water and blood. For CHCl_3_ from the bathing event, only the water-breath and blood-air correlations are significant. For BDCM, the significant correlations are those for blood and air and for blood and water from the shower activity.

The data presented in Table 4 suggest that the mean air and exhaled-breath concentrations, normalized with respect to the water concentrations, were different for the showering and bathing activities. For both CHCl_3_ and BDCM, the normalized mean air concentrations were much greater for the showering activity than for the bathing activity. In contrast, the breath concentrations for the showering and bathing activities were significantly smaller and essentially the same for the two activities. The ratios of the normalized mean breath to air concentrations were, however, much greater for the bathing activity than for the showering activity (0.37 vs. 0.08 for CHCl_3_; 0.40 vs. 0.11 for BDCM).

**Figure 4:**
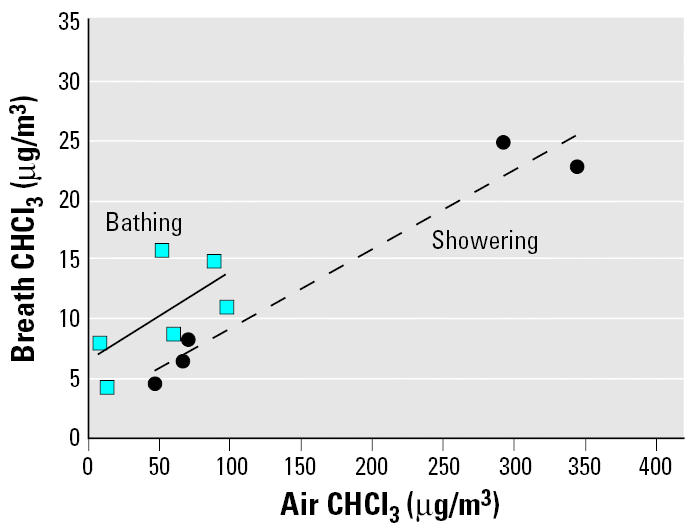
During-exposure air CHCl_3_ concentrations versus exhaled-breath (taken 5 min after exposure ended) CHCl_3_ concentrations from showering and bathing activities.

**Figure 5:**
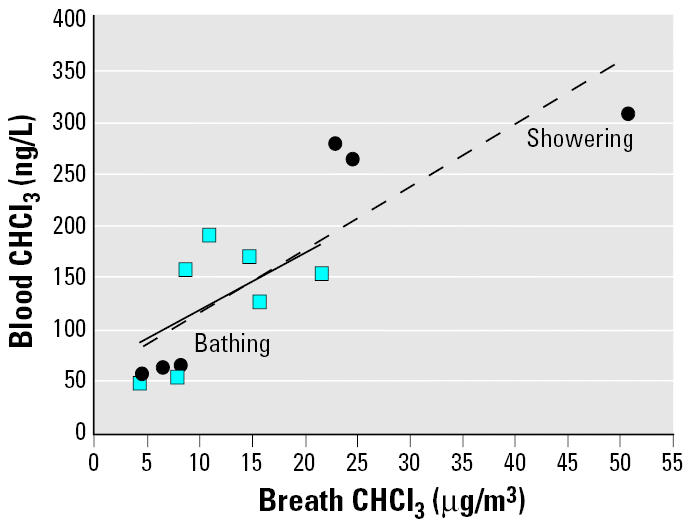
Exhaled-breath (taken 5 min after exposure ended) CHCl_3_ concentrations versus blood (taken 5 min after exposure ended) CHCl_3_ concentrations from showering and bathing activities.

Figure 4 shows plots of the CHCl_3_ concentrations in exhaled breath taken 5 min after the bath or shower exposure ended versus the air concentrations measured during the exposure period. The breath concentrations clearly increase with increasing air concentrations for these two events, and the slopes of the plots are quite similar, with the breath levels for the bathing activity greater than those for the showering activity. Figure 5 presents the CHCl_3_ concentrations in exhaled breath versus the corresponding blood concentrations, both of which were taken 5 min after the bath or shower exposure ended. The breath concentrations and blood concentrations correlate closely for the two activities.

## Discussion

### Effect of common water-use activities on indoor air and exhaled-breath concentrations.

The primary purpose of this study was to examine which common household water-use activities result in an increase in exhaled-breath concentrations and, hence, the internal dose of THMs in people conducting these activities. The results presented in Figure 2 for CHCl_3_ show that the showering and secondary shower exposure activities, as well as bathing, postsecondary shower exposure, and exposure due to mechanical washing of clothes with bleach resulted in appreciable increases in air concentrations for all subjects at both sampling sites, despite the large difference in tap-water CHCl_3_ concentration between the two sites.

Although much lower than the corresponding air levels, the mean normalized post-exposure breath concentrations of CHCl_3_ were consistently higher than the initial pre-exposure levels for the 10-min showering activity (mean ± SE, 0.19 ± 0.02 vs. 0.04 ± 0.01 μg/m^3^ per μg/L) and the 14-min bathing activity (0.25 ± 0.04 vs. 0.07 ± 0.03 μg/m^3^ per μg/L). Despite lower postexposure air and breath concentrations of BDCM than those obtained for CHCl_3_, the air concentrations associated with the showering, secondary shower exposure, and bathing activities were markedly higher than their preexposure concentrations. Only the showering and bathing activities resulted in moderately higher post-exposure breath concentrations of BDCM. As a result of the much lower DBCM and bromoform concentrations in the tap water in this study, neither chemical gave measurable breath concentrations for any of the water-use activities investigated (Nuckols et al. 2005).

There have been numerous laboratory-based studies of controlled exposure to THMs, especially CHCl_3_, from showering, bathing, or other household activities such as using a dishwasher (Andelman 1985; Gordon et al. 1998; Jo et al. 1990a, 1990b; Olson and Corsi 2004; Weisel and Jo 1996; Weisel et al. 1992). Those few that have been conducted in normal residential settings have been restricted to one or two common water-use activities and to measurements of CHCl_3_ concentrations in indoor air or exhaled breath. Table 5 shows that our observed normalized indoor air and exhaled-breath levels of CHCl_3_ and BDCM as a result of showering are consistent with results reported in previous residential exposure studies (Egorov et al. 2003; Kerger et al. 2000; May et al. 1995). Although the tap-water concentrations during showering vary markedly across the four studies cited in Table 5 (CHCl_3_, 47–198 μg/L; BDCM, 7–42 μg/L), the mean normalized indoor air concentrations during showering are quite similar (CHCl_3_, 1.67–3.52 μg/m3 per μg/L; BDCM, 1.25–1.91 μg/m^3^ per μg/L). The simple normalization procedure applied in the present study, and to the data from the three previous residential studies summarized in Table 5 (Egorov et al. 2003; Kerger et al. 2000; May et al. 1995), indicates generally good agreement between the studies. It should, however, be noted that in our study, the showering exposure conditions, and therefore the extent of volatilization, were uniform for all subjects.

The release of CHCl_3_ and BDCM during showering activities, expressed in terms of the mean normalized air concentrations in Table 5, shows a consistent concentration gradient of CHCl_3_ > BDCM. From our data, the mean normalized air concentration ratio (*C*_air_:*C*_water_) for CHCl_3_:BDCM is 1.17, which is within the range of values obtained for the other residential studies listed in Table 5 (1.12–1.84). To determine whether our measured concentration ratio is consistent with the physical properties of these compounds, we used a simple equilibrium model for predicting concentrations of VOCs in shower stall air (Sanders 2002). In this model, the entire mass of each chemical entering the system during the shower event is assumed to partition between the water and air phases under equilibrium conditions; loss of compound from the shower system by air exchange is ignored. All of the VOCs transferred from the water to the air phase during the showering event are assumed to remain in the shower stall. From the model, which is expressed in terms of a temperature-adjusted, dimensionless Henry’s law constant, *H*, and site-specific parameters, the ratio of the normalized concentrations of CHCl_3_ and BDCM in the air compartment at equilibrium is given by


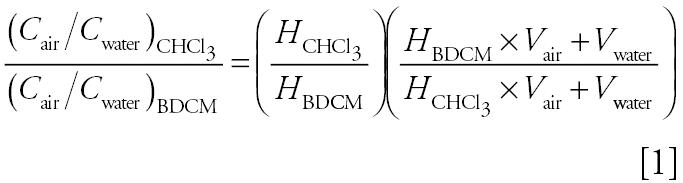


where *V*_air_ is the volume of the shower stall, and *V*_water_ is the total volume of water used during a shower event.

We used average values for the site-specific parameters, namely, 1.74 m^3^ for the volume of the shower stall, 0.08 m^3^ for the volume of the shower water (10-min shower duration with 8.0 L/min flow rate), no air exchange, and a shower water temperature of 41°C for calculating the dimensionless Henry’s law constants (Sander 1999; U.S. EPA 2005) for CHCl_3_ and BDCM. From Equation 1, the calculated normalized air concentration ratio for CHCl_3_:BDCM is 1.166, in excellent agreement with the experimentally measured ratio. This suggests that the simple equilibrium model may be used to predict the concentration ratios of THMs in shower systems and obtain insights into the behavior of those brominated THMs for which we have little or no data.

Published reports on the measurement of THM breath concentrations from exposures in residential settings are sparse. Table 5 includes our mean normalized breath concentration data for CHCl_3_ and BDCM along with values reported recently by Egorov et al. (2003). Although both data sets show similar concentration gradients from CHCl_3_ to BDCM as was found for shower air, our CHCl_3_ and BDCM breath concentrations and the CHCl_3_:BDCM ratio are markedly lower than the values obtained by Egorov et al. (2003). Two important reasons for these differences are the shower duration and the time when the samples were taken relative to the end of the exposure period. In our study, participants showered for 10 min, whereas participants in the Egorov et al. (2003) study showered for 15–20 min. The shorter showering period in our study would have resulted in a decrease in the air levels by as much as 50%. Additionally, previous work has shown that postexposure exhaled-breath concentrations of CHCl_3_ decrease rapidly with time, especially in the first few minutes after exposure (Gordon et al. 1998). The uptake and elimination residence times for BDCM are not known and are unlikely to be the same as those for CHCl_3_. Because breath samples were taken a full 5 min after exposure ceased in our study, whereas breath samples were collected within 1 min after the subjects completed their showering activity in the Egorov et al. (2003) study, differences in these factors may explain the large differences in the observed breath values in Table 5.

Other differences that may limit the comparability between studies in Table 5 include the presence of a minimal free chlorine residual and the lack of control over shower duration, flow rate, and temperature among the subjects from different households in the Egorov et al. (2003) study.

### Relationships between air, water, blood, and breath measurements.

We examined the relationships between the concentrations of CHCl_3_ and BDCM associated with both the showering and bathing activities in water, air, blood, and breath. The data in Table 4 indicate that the mean normalized air concentrations of CHCl_3_ and BDCM are much lower for the bathing event, probably due to the lesser opportunity for gas transfer between the bath water and the air. In contrast, the higher volatilization rate of CHCl_3_ and BDCM into the shower stall air from the shower spray was the likely reason for the significantly higher air levels observed for the showering activity.

Despite the higher CHCl_3_ and BDCM air concentrations noted in Table 4 for the showering activity than for the bathing activity, the respective breath concentrations are essentially the same. This suggests that the overall rate of uptake of the two THM species is roughly the same and that the dermal route is the more important exposure pathway (vs. inhalation) in the bath than in the shower. Further confirmation of the importance of the dermal route is provided by a comparison of the effect of secondary shower exposure on the measured air and breath concentrations (Figure 2). The large increase observed in the air concentration with secondary shower exposure almost matches, not surprisingly, that for the showering activity. However, contrary to the role of the showering activity in raising the breath concentration, the elevated air concentration due to the secondary shower exposure had no such effect on the breath concentration.

The relatively strong correlations between blood CHCl_3_ and breath CHCl_3_ concentrations suggested by the Spearman correlation coefficients for the showering and bathing activities are further supported by the plots in Figure 5. The association derives from the dynamic equilibrium that is assumed to exist between the concentration of the volatile compound in arterial blood and its concentration in alveolar breath (Wallace et al 1996). Thus, the breath concentration can be used as a proxy to estimate body burden or changes in body burden with time (Gordon et al. 1992; Raymer et al. 1991; Wallace et al. 1993; Weisel et al. 1992). The slopes of the regression lines in Figure 5 may be used to estimate the average venous blood-to-breath ratio for CHCl_3_. The average CHCl_3_ blood-to-breath ratios from the showering and bathing activities were estimated to be 6.9 (*R*^2^ = 0.84) and 7.5 (*R*^2^ = 0.61), respectively, which compare favorably with the blood/air partition coefficient (6.9) reported by Gargas et al. (1989), but are smaller than the value (10.7) reported more recently by both Fisher et al. (1997) and Batterman et al. (2002).

In summary, we found that showering and bathing are two common household water-use activities that cause significant increases in exhaled breath concentrations of CHCL_3_. Further analysis of the data for CHCL_3_ from showering indicated strong correlations for indoor air and exhaled breath, blood and exhaled breath, indoor air and blood, and tap water and blood. For CHCL_3_ from bathing, only water and breath, and blood and breath showed strong associations. Future studies are warranted to explore changes in these activities that can affect exposure.

## Figures and Tables

**Table 1:** Summary of monitored water-use activities.

Day	Water-use activity
1	1. Baseline measurements before any water use
	2. Drink hot beverage (0.25 L)
	3. Hot-water shower^a^
	4. Drink cold tap water (0.5 L)
	5. Automatic clothes washing^b^
	6. Hand washing
	7. Automatic dishwashing
	8. Open/remove dishes from dishwasher at end of cycle
2	1. Baseline measurements before any water use
	2. Drink cold water-based (fruit juice) beverage (0.25 L)
	3. Hot-water bath
	4. Automatic clothes washing; bleach added during wash cycle^b^
	5. Hand washing of dishes
	6. Subject stayed in room adjoining study bathroom during shower (secondary shower exposure)^c^
	7. Subject in room adjoining study bathroom after shower (postsecondary shower exposure)^c^

a Five subjects followed the standard shower protocol; two subjects undertook the intensive shower protocol.

b Participant did not stay in same room while washing machine was operating.

c Bathroom door was opened at end of shower event.

**Table 2:** Overall mean water concentrations (μg/L) for CHCl_3_ and BDCM over all subjects by exposure day and location.

		Water concentration (mean ± SD)	
Location	Exposure day^a^	CHCl_3_	BDCM
NC	1	126.2 ± 14.4	31.9 ± 11.4
	2	90.4 ± 6.4	24.4 ± 7.7
TX	1	28.5 ± 4.1	10.6 ± 2.8
	2	22.2 ± 2.5	8.6 ± 2.0

a Exposure days were 1 week apart.

**Table 3:** Spearman rank correlations among air and water exposure measurements and breath and blood biomarkers in shower and bath samples from all subjects.

	Air		Breath		Blood	
Analyte (medium)	CHCl_3_	BDCM	CHCl_3_	BDCM	CHCl_3_	BDCM
Hot shower						
CHCl_3_ (air)	—	—	0.94*	—	0.99**	—
BDCM (air)	—	—	—	0.61	—	0.94*
CHCl_3_ (water)	0.83	—	0.90	—	0.82*	—
BDCM (water)	—	0.83	—	0.75	—	0.82*
CHCl_3_ (blood)	—	—	0.94*	—	—	—
BDCM (blood)	—	—	—	0.53	—	—
Hot bath						
CHCl_3_ (air)	—	—	0.54	—	0.93**	—
BDCM (air)	—	—	—	0.54	—	0.64
CHCl_3_ (water)	0.54	—	0.94*	—	0.71	—
BDCM (water)	—	0.60	—	–0.37	—	0.14
CHCl_3_ (blood)	—	—	0.43	—	—	—
BDCM (blood)	—	—	—	0.14	—	—

* *p* < 0.05.

** *p* < 0.005.

**Table 4:** Normalized mean (± SE) air and exhaled-breath concentrations (μg/m^3^ per μg/L) for CHCl_3_ and BDCM over all subjects from showering and bathing activities.

	Air		Breath	
THM component	Showering	Bathing	Showering	Bathing
CHCl_3_	2.28 ± 0.20	0.68 ± 0.12	0.19 ± 0.02	0.25 ± 0.04
BDCM	2.09 ± 0.27	0.55 ± 0.10	0.23 ± 0.05	0.22 ± 0.06

**Table 5:** Comparison of normalized CHCl_3_ and BDCM concentrations in tap water, during-shower air, and postshower breath from THM exposure studies.

	THM concentration^a^	
Target matrix	CHCl_3_	BDCM
Mean water concentration (μg/L)^b^		
May et al. (1995)^c^	55	17
Kerger et al. (2000)^d^	47	42
Egorov et al. (2003)	198	6.7
This study	82.8	20.3
Mean normalized during-shower air concentration (μg/m^3^ per μg/L)^e^		
May et al. (1995)^c^	1.72	1.54
Kerger et al. (2000)^d^	3.52	1.91
Egorov et al. (2003)	1.67	1.25
This study	2.23 ± 0.18	1.91 ± 0.23
Mean normalized postshower breath concentration (μg/m^3^ per μg/L)^f^		
May et al. (1995)^c^	NA	NA
Kerger et al. (2000)^d^	NA	NA
Egorov et al. (2003)	0.54	0.12
This study	0.15 ± 0.01	0.07 ± 0.02

NA, not analyzed.

a In water source, bromoform was near or below LOD at most sites; in air samples, DBCM and bromoform were < LODs in Egorov et al. (2003) and May et al. (1995) studies; in breath samples, DBCM and bromoform were < LODs in Egorov et al. (2003) study and this study.

b *n* = 44 in May et al. (1995) study; *n* = 20 for source water and *n* = 12 for shower air in Kerger et al. (2000) study; *n* = 14 for source water, *n* = 35 for shower air, and *n* = 9 for exhaled breath in Egorov et al. (2003) study; and *n* = 6 for source water, shower air, and exhaled breath in this study.

c Data for mean values for CHCl_3_ and BDCM for source water and shower air estimated from plots in May et al. (1995).

d Air concentration data obtained by Kerger et al. (2000) from eight unvented and four vented shower events. In vented events, either the bathroom exhaust fan was on or the bathroom window was opened during the sampling event. The bathroom door was shut for all shower sampling events.

e Shower duration: May et al. (1995) reported 10 min; Kerger et al. (2000) reported 6.8 min and 12 min; Egorov et al. (2003) reported 15–20 min; this study, 10 min.

f Breath sample collection: Egorov et al. (2003) reported ≤1 min postexposure; this study, 5 min postexposure.
